# Comparative analysis of uranium bioassociation with halophilic bacteria and archaea

**DOI:** 10.1371/journal.pone.0190953

**Published:** 2018-01-12

**Authors:** Miriam Bader, Katharina Müller, Harald Foerstendorf, Matthias Schmidt, Karen Simmons, Juliet S. Swanson, Donald T. Reed, Thorsten Stumpf, Andrea Cherkouk

**Affiliations:** 1 Helmholtz-Zentrum Dresden—Rossendorf, Institute of Resource Ecology, Dresden, Germany; 2 Helmholtz Centre for Environmental Research - UFZ, Department of Isotope Biogeochemistry, Leipzig, Germany; 3 Los Alamos National Laboratory Carlsbad Operations, Repository Science and Operations, Carlsbad, New Mexico, United States of America; Centre for Research and Technology-Hellas, GREECE

## Abstract

Rock salt represents a potential host rock formation for the final disposal of radioactive waste. The interactions between indigenous microorganisms and radionuclides, e.g. uranium, need to be investigated to better predict the influence of microorganisms on the safety assessment of the repository. Hence, the association process of uranium with two microorganisms isolated from rock salt was comparatively studied. *Brachybacterium* sp. G1, which was isolated from the German salt dome Gorleben, and *Halobacterium noricense* DSM15987^T^, were selected as examples of a moderately halophilic bacterium and an extremely halophilic archaeon, respectively. The microorganisms exhibited completely different association behaviors with uranium. While a pure biosorption process took place with *Brachybacterium* sp. G1 cells, a multistage association process occurred with the archaeon. In addition to batch experiments, *in situ* attenuated total reflection Fourier-transform infrared spectroscopy was applied to characterize the U(VI) interaction process. Biosorption was identified as the dominating process for *Brachybacterium* sp. G1 with this method. Carboxylic functionalities are the dominant interacting groups for the bacterium, whereas phosphoryl groups are also involved in U(VI) association by the archaeon *H*. *noricense*.

## Introduction

Rock salt is a potential host rock for a final repository of radioactive waste [1]. Many microorganisms are indigenous to rock salt and are able to survive under these extreme environmental conditions, namely high salinities and the lack of nutrients [2–4]. These microorganisms can influence the mobility of radionuclides should the radionuclide be released in a worst-case scenario. Possible bioassociation interactions include the following processes [5–7]: bioaccumulation (uptake of the radionuclide by the cell), biotransformation (change to the oxidation state of the radionuclide), biomineralization (formation of an insoluble precipitate containing the radionuclide and a compound released by the microbes) and biosorption (passive binding of the radionuclide to functional groups of living or dead biomass).

Halophilic microorganisms can be classified as moderate halophiles, which require 0.5 – 2.5 M NaCl, and extreme halophiles, which require 2.5 – 5.2 M NaCl [8–10]. Even though Bacteria as well as Archaea have halophilic members, the extreme halophiles belong commonly to Archaea [8]. Nevertheless, it is important to include thermodynamics and interaction mechanisms of all possible microorganisms in the safety assessment of a potential repository. Only a few studies have been reported regarding the investigation of halophilic microorganisms with radionuclides [11–14]. A study by Francis and coworkers [12] compared the interactions of halophilic archaea and bacteria, as well as non-halophilic bacteria, with uranium. Transmission electron microscopy (TEM) images showed that uranium bound to the cell surface of all investigated microorganisms. Additionally, intracellular granules were observed within the bacterium *Halomonas* (WIPP A1). However, there is still little knowledge available about the molecular interactions of radionuclides with microorganisms under highly saline conditions and as function of time.

The composition of the microbial cell wall is crucial for a passive biosorption process. The cell envelopes of Archaea and Bacteria are entirely different. Archaea lack a universal cell wall polymer and have a different lipid composition than Bacteria, the isoprenoid side chains are ether-linked, whereas bacterial are mostly ester-linked [15]. Instead of the bacterial rigid cell wall component peptidoglycan (murein), a crystalline protein layer on the cell surface is present for most archaea [15]. Early studies with the extremely halophilic archaeon *Halobacterium salinarum* (formerly named *Halobacterium halobium*) showed the presence of such a surface layer (S-layer) protein [16]. This was also found for *Halobacterium noricense* DSM15987^T^, the strain of our interest [17]. The S-layer proteins of Haloarchaea are glycosylated and are adapted to high salinity, e.g. they have an enriched amount of acidic residues [18]. The moderately halophilic bacterium used in this work, *Brachybacterium* sp. G1, belongs to Gram-positive bacteria. The outer phase of the cell wall is composed of peptidoglycan layers with anchored molecules, such as sugars and amino acids. For *Brachybacterium faecium* DSM4810^T^, the sugars galactose, glucose and glucosamine are present [19, 20].

In this study, we investigated the bioassociation of *Brachybacterium* sp. G1, an isolate of the German salt dome Gorleben, with uranium as a function of time, uranium concentration and dry biomass (DBM) concentration. Studies were performed at a pH value of 5.5, which is a typical pH for brines analyzed from US and German subterranean salt formations [21, 22]. Due to its high inventory in nuclear waste, e.g. through production of enriched nuclear fuel or as a by-product of spent-fuel, uranium was used as a representative radionuclide in this study [23]. Under oxic conditions U(VI) is the dominant oxidation state, which is highly soluble. Whereas under reducing conditions U(IV) is dominant and immobile, favoring the retardation of this radionuclide [24]. In addition to microbially induced reduction, the formation of insoluble minerals by microorganisms (biomineralization) is another possible retention process. The formation of uranyl phosphate minerals has been commonly observed [25–27].

The localization of uranium was visualized by electron microscopy, and cell viability was verified by fluorescence microscopy. Furthermore, we used *in situ* attenuated total reflection Fourier-transform infrared spectroscopy (ATR FT-IR) to elucidate the bioassociation process at a molecular level within a timeframe of 2 h. The obtained results regarding the bioassociation of uranium by *Brachybacterium* sp. G1 were compared with the results from the extremely halophilic archaeon *H*. *noricense* [11].

## Materials and methods

### Isolation of *Brachybacterium* from a Gorleben rock salt sample

**Fig 1:**
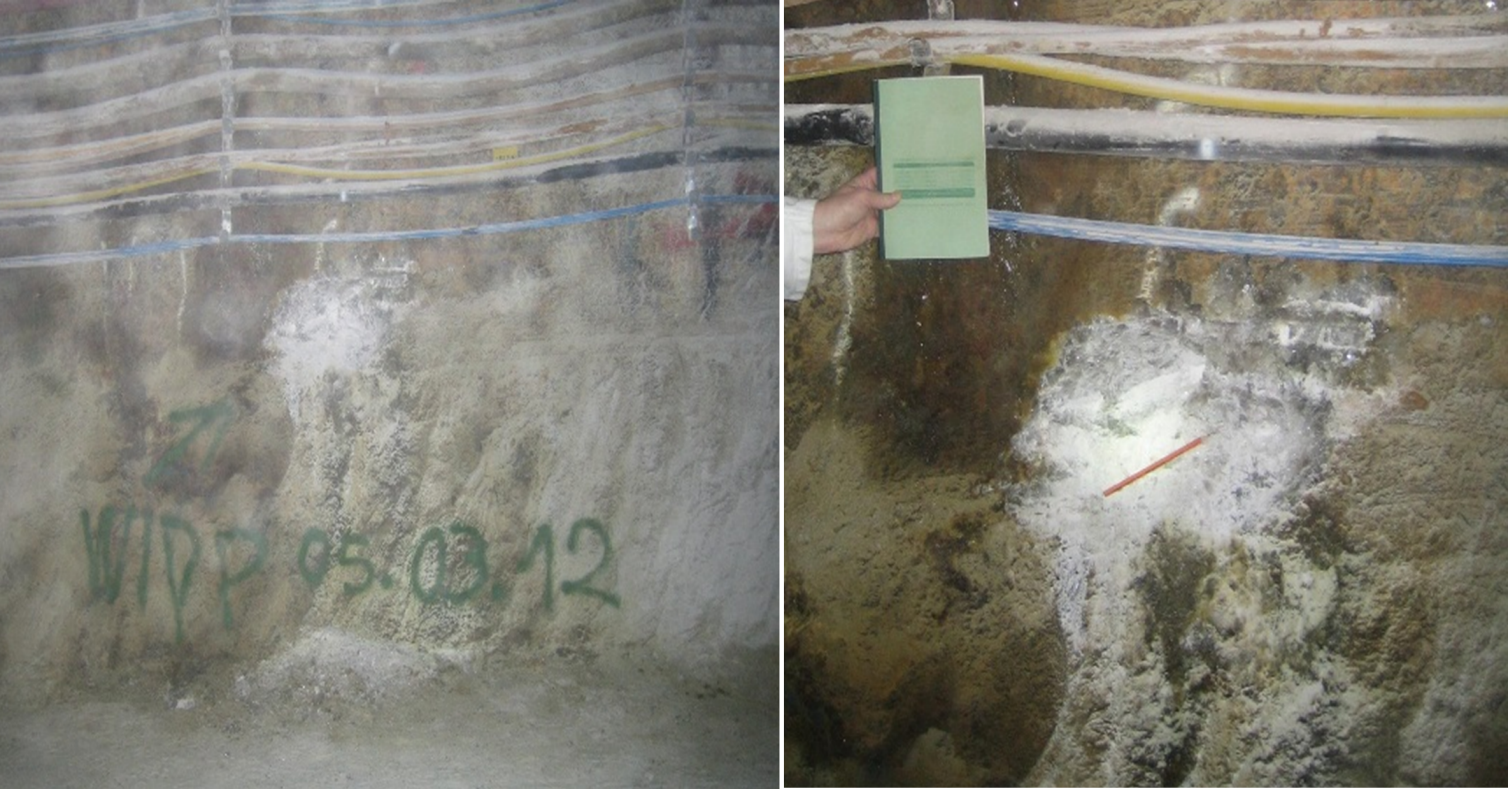
Sampling area from the Gorleben halite in a depth of 840 m within the Knäulsalz layer.

Gorleben halite was retrieved from a depth of 840 m within the Knäuelsalz layer, in an area known to contain a high concentration of total hydrocarbons - up to 400 ppm C_1_-C_40_ and aromatic substances [28]. A significant green biofilm was removed from the sampling area prior to the aseptic removal of halite samples from underneath (Fig 1). Samples were triple-sealed in sterile bags and stored in the dark at ambient temperature until processing in the laboratory.

Approximately 50 g of the hydrocarbon-containing halite was inoculated into 500 mL of low-nutrient medium (per L: 0.5 g yeast extract, 0.5 g casamino acids, 20 g MgCl_2_·6H_2_O, 2 g KCl, 0.2 g CaCl_2_·2 H_2_O, 0.015 g sodium pyruvate, 1.1 mL of 1 M stock Tris-HCl, pH 8.5, 1 mL American Type Culture Collection trace elements) and incubated aerobically at 22 ± 2°C [29]. The halite sample was the only form of sodium chloride added to the medium for this incubation, resulting in approximately 10% (w/v) NaCl in solution. Growth in the inoculated sample was observed within 3 weeks. Control samples remained sterile. The samples were subcultured onto SW-10 agar [9], yielding growth of yellow-pigmented colonies. The 16S ribosomal RNA gene was amplified with an *in situ* PCR, using the primers 7f and 1513r (initial denaturation at 95°C for 3 min; followed by 5 cycles initial amplification of 95°C denaturation (1.5 min), 59°C annealing (40 s, in each of the 5 cycles a temperature gradient of -1°C), and 72°C elongation (1.5 min); followed by 25 cycles of 94°C denaturation (1 min), 55°C annealing (40 s) and 72°C elongation 1.5 min, and a final 20 min elongation at 72°C). Amplicons were purified with the QuickStep^TM^2 PCR Purification kit (EdgeBio, MD, USA) prior to submission to GATC Biotech AG (Cologne, Germany) for sequencing. The 16S_7f_ and 16S_1513r_ sequences were aligned using MEGA 6.06 software (ClustalW) [30] and compared to those from GenBank using the Basic Local Alignment Search Tool (BLAST) server at the National Center for Biotechnology Information (NCBI) [31]. A phylogenetic dendrogram was generated with MEGA 6.06 [30] using the neighbor-joining algorithm and was validated by bootstrap analysis using 500 trial replicates.

To determine the DBM, cells with a defined optical density were pelleted in previously dried (48 h, 70°C) and weighed tubes, dried for 48 h at 70°C and weighed. This determination was made in triplicate.

### Cultivation of the microorganisms

*Brachybacterium* sp. G1. The moderately halophilic bacterium was cultivated in Difco^TM^ Marine Broth 2216 (Becton, Dickinson and Company, USA) adding NaCl to a final concentration of 10% (1.7 M). The media were filter-sterilized (pore size 0.22 μm) after autoclaving to remove precipitates. Cell suspensions were shaken at 140 rpm at room temperature. The growth was investigated as a function of different parameters. The pC_H+_ dependency was studied in a range of 3.2–10.7 at a constant NaCl concentration of 4.5% (0.8 M). Salt-dependent studies were performed at pC_H+_ 7.6 ± 0.2, whereas the ionic strength was varied from 0 to 5.1 M using different ratios of NaCl and MgCl_2_.

*Halobacterium noricense*. The extremely halophilic archaeon *H*. *noricense* DSM15987^T^ was purchased from the Leibniz Institute DSMZ - German Collection of Microorganisms and Cell Cultures (DSMZ, Braunschweig, Germany). It was cultivated in modified DSM372 medium [11]. During growth, the cultures were shaken at 140 rpm in the dark in a water bath at 30°C.

Cells were harvested during late exponential growth by centrifugation at 10,000 *x g* and 18°C for 10 min and washed three times with the appropriate NaCl solution (3 M NaCl for *H*. *noricense* / 1.7 M NaCl for *Brachybacterium*) at pC_H+_ 6 to prepare cells for batch studies (see below).

### pH correction at high salinity

Due to the high content of salt, the measured pH (negative logarithm of the hydrogen ion activity) had to be corrected. Therefore, the term pC_H+_, as hydrogen ion concentration, was introduced. According to the empirical equation of Borkowski *et al*. [32], the relationship between pC_H+_ and the measured pH is a linear function of the ionic strength:

$$pC_{H+}=pH\;measured+K$$ (1)

K is linear to ionic strength:

K=(0.1868xI)–0.073 (2)

For a 3 M NaCl solution K is 0.49, for a 1.7 M NaCl solution the pH has to be corrected by adding a K of 0.24.

### Uranium bioassociation in batch systems

For uranium bioassociation studies, washed cells from the late exponential growth phase were used. A defined amount of *Brachybacterium* sp. G1 cells, expressed as DBM, were suspended in 5 mL of 1.7 M NaCl solution at pC_H+_ 6 and diluted with 5 mL uranium solution, containing 1.7 M NaCl at pC_H+_ 6. The uranium stock solution was prepared by dissolving UO_2_(NO_3_)_2_ x 6 H_2_O (Spectrum Chemical Mfg. Corp., USA) in 1 M HCl and heating it to dryness three times. It was then precipitated with sodium hydroxide and dissolved in 0.2 M HCl to a final concentration of 230 mM U(VI). Depending on the experiment, the uranium concentration ranged from 10 – 120 μM. At the investigated pC_H+_ value, the dominant species is the 3:5 uranyl hydroxo complex—(UO_2_)_3_(OH)_5_^+^ [33]. For batch experiments DBM concentrations from 0.075–0.125 g/L were used. They were carried out under aerobic conditions in 50 mL tubes, at 25°C in the dark and shaken at 150 rpm. First, time-dependent bioassociation studies with U(VI) were performed over time frames of 5 min up to 48 h. Later, for uranium as well as DBM concentration dependence experiments, an exposure time of 2 h was used. After the designated exposure time, a 500 μL aliquot of a cell suspension of *Brachybacterium* sp. G1 was filtered with 100 kD Amicon^®^ 2 mL centrifugal filters (Merck Millipore) for 15 min at 13,000 *x g*. The concentration of unbound uranium in the filtrate was measured with ICP-MS (Inductively coupled plasma mass spectrometry, Agilent 7500ce; Agilent Technologies, Santa Clara, CA, USA). The experiments with *Brachybacterium* sp. G1 were performed at CEMRC, NM, USA, whereas studies with *H*. *noricense* DSM15987^T^ were done at HZDR.

Therefore, the bioassociation studies with *H*. *noricense* DSM15987^T^ were slightly modified. After washing, a defined amount of *H*. *noricense* cells was pelleted and resuspended in 10 mL uranium solution, pC_H+_ 6, containing 3 M NaCl [11]. After exposure, the suspension was centrifuged (18°C, 10,000 *x g*, 10 min). For analyzing the amount of unbound uranium, a sample from the supernatant was analyzed by ICP-MS (Elan 9000 Perkin Elmer, Waltham, MA, USA). Cells were washed with 3 M NaCl, pC_H+_ 6 for further experiments.

The amount of bound uranium was normalized to DBM or reported as the percentage of added uranium. Control samples without cells were treated in the same way such that abiotic uranium removal from the solution, due to precipitation and/or chemical sorption to the vials could be excluded. Except when otherwise stated, experiments were performed as triplicates and reported errors are standard deviations.

### Verification of cell viability

Cell suspensions (1 mL) treated under different experimental conditions were centrifuged at 18°C and 10,000 *x g* for 10 min. For Live/Dead staining the Kit LIVE/DEAD® BacLight^TM^ Bacterial Viability Kit L7012, (Molecular Probes^TM^ Inc., Eugene, OR, USA) was used according to the manufacturer´s instructions. The samples were analyzed with an Olympus BX-61 (Olympus Europa Holding GmbH, Hamburg, Germany) microscope using the imaging software ‘‘cellSense Dimension 1.11”. Fluorescence was excited by light with wavelengths between 420 and 460 nm using a super wideband filter mirror unit. The suitability of this kit for halophilic microorganisms was demonstrated by Leuko *et al*. [34].

### Scanning electron microscopy coupled with energy dispersive X-ray spectroscopy

The localization of U(VI) on cells of *Brachybacterium* sp. G1 was determined using scanning electron microscopy (SEM) coupled with energy dispersive X-ray (EDX) spectroscopy for elemental mapping. Cells exposed to 20 μM U(VI) for 2 h and 48 h were dried on a silicon wafer at room temperature. No fixation steps were performed to avoid preparation-induced changes. The samples were analyzed at ProVIS, Centre for Chemical Microscopy at the Helmholtz Centre for Environmental Research (Leipzig, Germany) with a scanning electron microscope (Zeiss Merlin VP Compact, Carl Zeiss Microscopy, Germany) coupled with an EDX detector (Quantax X-Flash, Bruker Nano GmbH, Berlin, Germany). The electron-acceleration energy was set to 5.8 kV.

### *In situ* ATR FT-IR spectroscopy

The association processes of U(VI) on cells of *Brachybacterium* sp. G1 as well as *H*. *noricense* were monitored using *in situ* ATR FT-IR spectroscopy with a sub-minute time resolution [32, 33]. The application of reaction-induced difference spectroscopy allows the detection of very small absorption changes provoked by the bioassociation process despite the presence of a very strong absorbing background, i.e. water and microbial cell film.

The microbial cells were prepared as a thin film directly on the ATR crystal´s surface, which was a horizontal diamond crystal (A = 12.57 mm^2^, DURASamplIR II, Smiths Inc.) with nine internal reflections on the upper surface and a 45° angle of incidence. An aliquot of 7.5 μL of a freshly harvested and three times washed suspension of *H*. *noricense* DSM15987^T^ or *Brachybacterium* sp. G1 with a concentration of 0.5 g/L DBM was pipetted onto the crystal and dried under a gentle flow of nitrogen. This procedure was repeated.

For the *in situ* sorption experiment the cell film was at first equilibrated by flushing a blank solution (3 M NaCl for *H*. *noricense*, 1.7 M NaCl for *Brachybacterium* sp. G1, at pC_H+_ 6) for 60 min using a flow cell (V = 200 μL) at a rate of 200 μL/min (Equilibrium phase). Subsequently, the sorption reactions were induced by rinsing the microbial film for the next 120 min with a 40 μM U(VI) solution, equal in background electrolyte and pC_H+_ to the blank solution. In order to gain information on the reversibility of the process, the blank solution was flushed for another 60 min in a third step.

Infrared spectra were continuously recorded on a Bruker Vertex 80/v vacuum spectrometer, equipped with a Mercury Cadmium Telluride (MCT) detector with a low frequency cut-off at 600 cm^−1^. Spectral resolution was 4 cm^−1^ and spectra were averaged over 256 scans. Müller et al. [35, 36] give further details on the experiments performance and on the calculation of difference spectra.

## Results and discussion

### Phylogenetic characterization of the isolate

**Fig 2:**
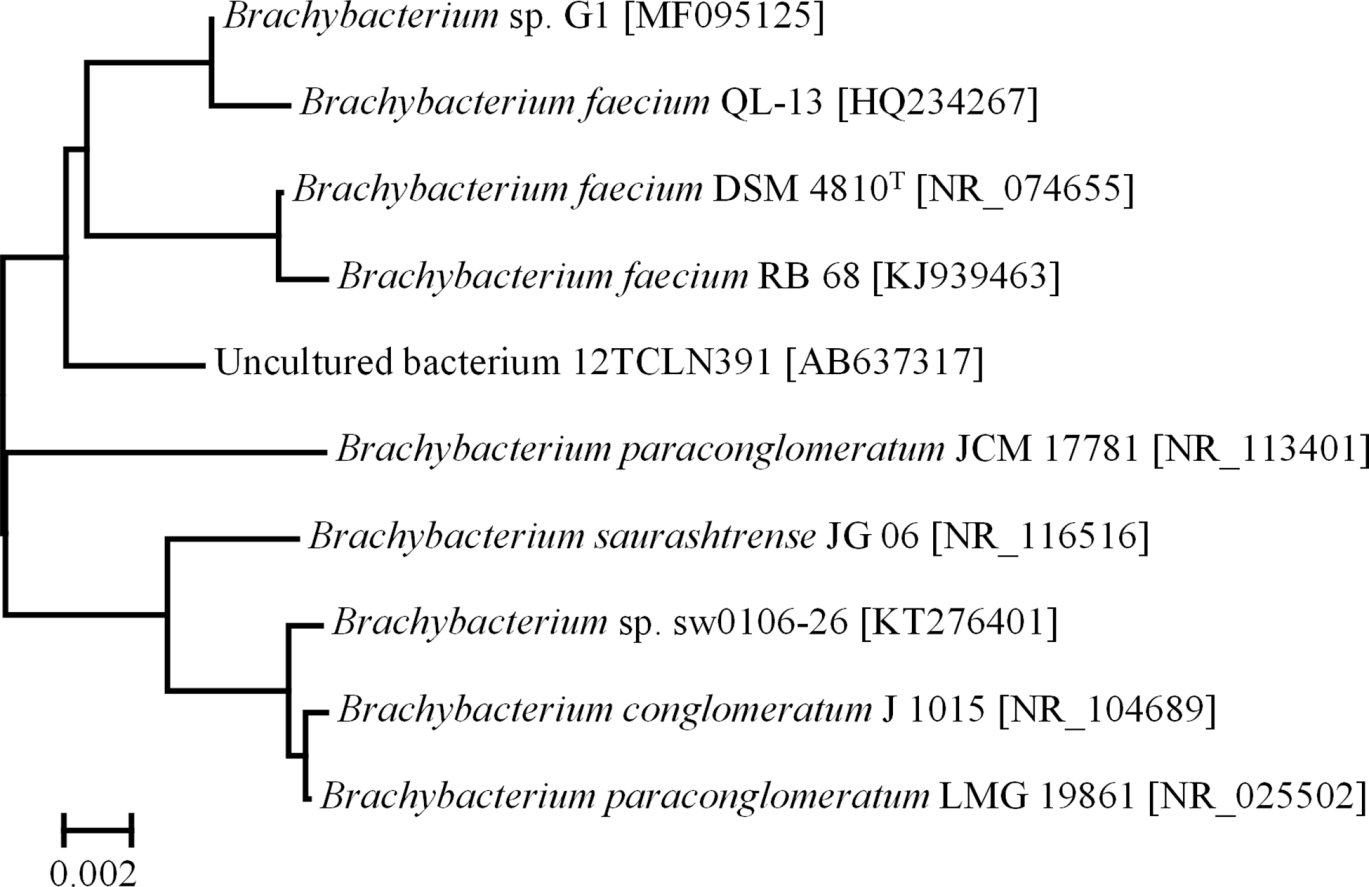
Phylogenetic dendrogram (neighbor-joining method) of *Brachybacterium* sp. G1 and its closest phylogenetic relatives based on an alignment of 16S rRNA gene sequences (aligned with ClustalX-MEGA 6.06). GenBank accession numbers are shown in brackets.

The obtained isolate *Brachybacterium* sp. G1 (Accession No. MF095125) showed 99% sequence similarity to the 16S rRNA gene sequence of *Brachybacterium faecium* strain QL-13 (Accession No. HQ234267), *Brachybacterium faecium* strain DSM4810^T^ (Accession No. NR_074655) and *Brachybacterium faecium* strain RB 68 (Accession No. AKJ939463) (Fig 2). *Brachybacterium faecium* strain QL-13 was isolated from a natural *Populus euphratica* of disused ancient Kiyik River Xinjiang in China and *Brachybacterium faecium* strain RB 68 was isolated from continental shelf sediments of the Arabian Sea. The type strain *Brachybacterium faecium* DSM4810^T^ was first isolated from poultry deep litter [37, 38]. Other *Brachybacterium* species were isolated for instance from Antarctic sea ice brine [39] and the Great Salt Lake, Utah [40].

### Characterization of the growth of the moderately halophile bacterium

**Fig 3:**
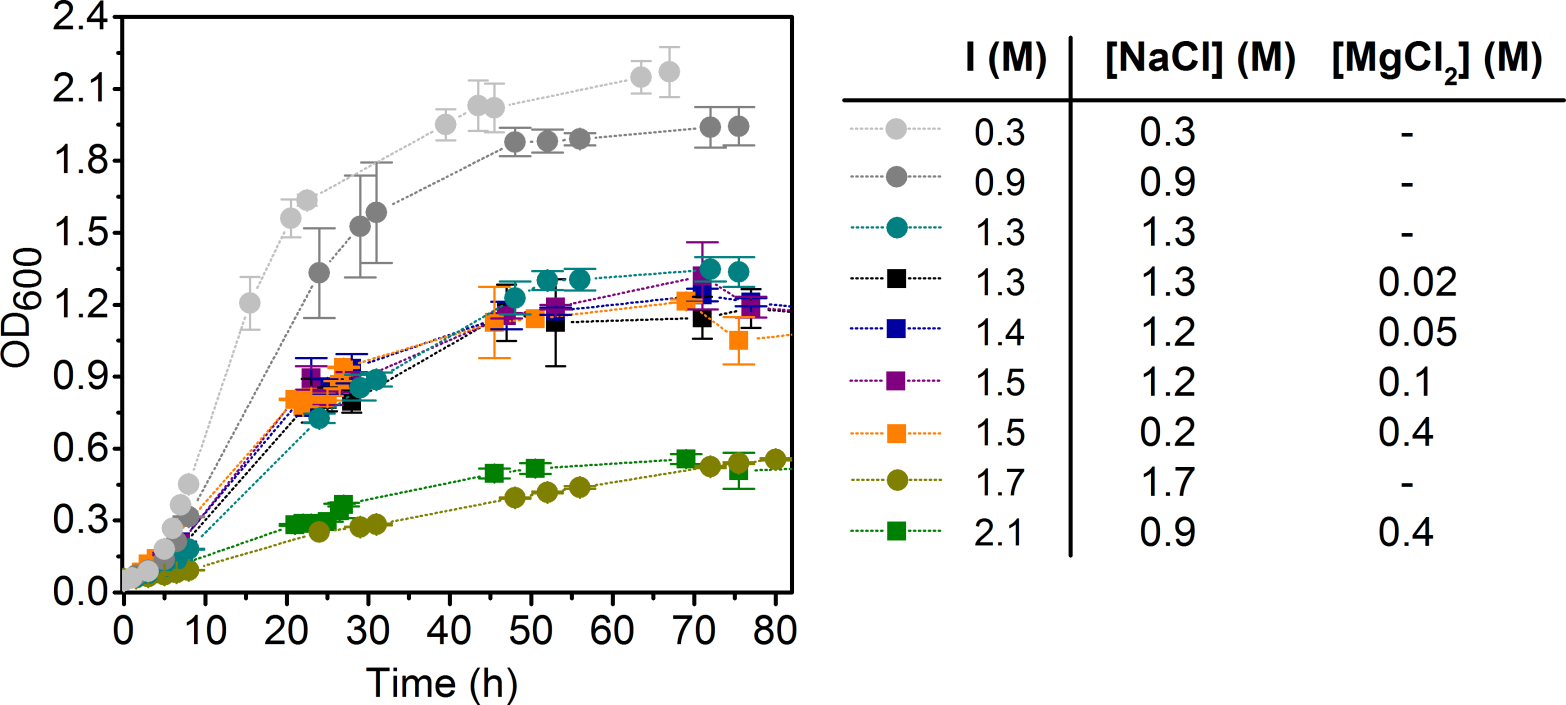
Growth of *Brachybacterium* sp. G1 as a function of ionic strength and salt composition. Circles indicate NaCl containing media, squares indicate the addition of MgCl_2_.

Growth studies on the *Brachybacterium* sp. G1 isolate showed a tolerance range of 0.3-2.1 M NaCl with an optimum growth between 0.3-1.3 M NaCl. No growth was observed for salt concentrations above 3.4 M NaCl. From variations of the NaCl / MgCl_2_ ratios it can be concluded that the total ionic strength is more relevant than the composition of the salt fraction. For instance, the same growth behavior was observed for ionic strength of 1.5 M having two different ratios of salt (Fig 3). *Brachybacterium* sp. G1 grew best with an ionic strength below 0.9 M. No differences occurred in the range of 1.3 M-1.5 M, but elevating the ionic strength to 1.7 M and higher led to an increased inhibition of growth. This suggests that *Brachybacterium* sp. G1 is a moderately halophilic bacterium [8, 10]. Junge and coworkers isolated a *Brachybacterium* sp. from Antarctic Sea ice brine and got similar results. The strain was able to grow at relatively high salinities but the presence of salt was not essential, supporting the assumption that they were physiologically adapted to grow at high salt concentrations. This was explained either by a slow adaption from a low salt containing environment or the growth within a various salt containing environment as a selective advantage [39]. The strain is moderately alkaliphilic with a somewhat restricted pC_H+_ range between pC_H+_ 7.1–9.1, whereas the optimum is pC_H+_ 8.1 and minimal growth occurs at pC_H+_ values 6.1 and 10.1 (S1 Fig). Like other *Brachybacterium* species [19, 37], *Brachybacterium* sp. G1 exhibits two morphologies: large cocci occurring in pairs, tetrads, or clumps and short rods in early exponential phase.

### Uranium bioassociation behavior

**Fig 4:**
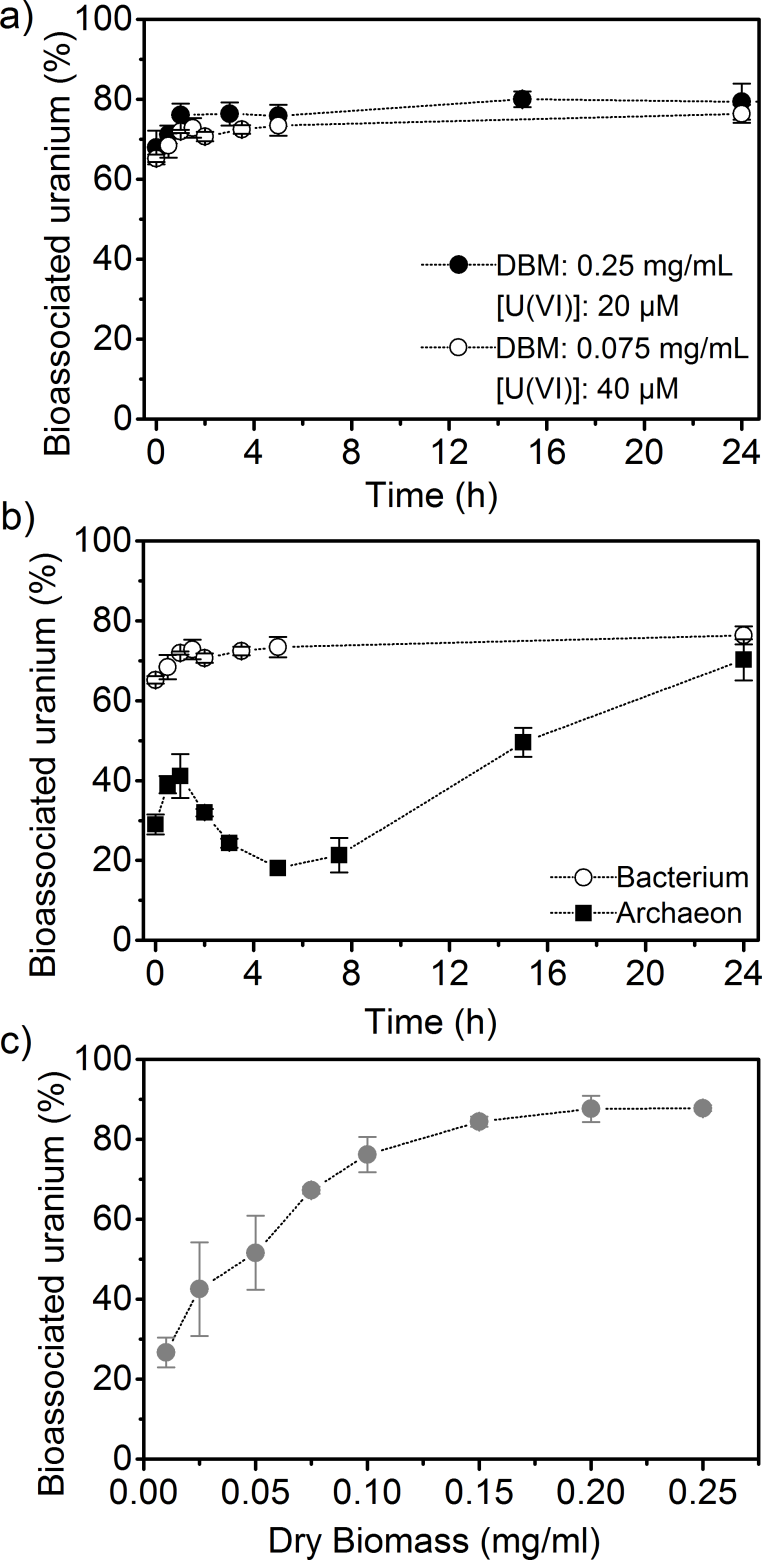
Bioassociation studies with *Brachybacterium* sp. G1 and uranium (pC_H+_ 6, 1.7 M NaCl). a) Time-dependent association with different dry biomass and uranium concentrations, b) comparison to *H*. *noricense* (3 M NaCl, 0.5 mg/mL DBM) [11] at 40 μM, c) dry biomass dependent study [U(VI)] = 20 μM).

The uranium bioassociation behavior by cells of *Brachybacterium* sp. G1 was studied as a function of different parameters to gain insights into the interactions of radionuclides with halophilic bacteria. The parameters tested were exposure time, DBM and uranium concentration. Their impacts are summarized in Fig 4A. No differences in bioassociation behavior were apparent when varying either DBM (0.25 mg/mL and 0.075 mg/mL) or uranium concentrations (20 and 40 μM). The high bioassociation values obtained at low DBM (0.075 mg/mL) and increased uranium concentration (40 μM) demonstrate the high sorption capacity of *Brachybacterium* towards uranyl species. Under these conditions, the amount of U(VI) associated with the bacterium increased from 870 ± 40 mg_U_/g_DBM_ after 1 h to 971 ± 29 mg_U_/g_DBM_ after 24 h of exposure. This fast association is characteristic for a biosorption process, which refers to a metabolism-independent association of a sorbate, in our case uranium, to biomass [5, 41] and has been reported for a number of bacteria [42, 43]. Another Actinobacteria, *Arthrobacter simplex*, adsorbed only 58 mg_U_/g_DBM_ under slightly different conditions (pH 4.6, 42 μM U(VI), 0.05 mg/mL DBM, 30°C) [43]. In contrast to the simple biosorption process, the bioassociation behavior of uranium (40 μM) with the archaeon *H*. *noricense* was found to be different [11] (Fig 4B). *H*. *noricense* association with uranium was a rather complex process including multiple steps. Within the first hour of exposure a typical biosorption was observed, followed by a release of uranium for the next four hours and eventually a biomineralization process [11]. Such a multistage uranium bioassociation process was also seen for *Microbacterium* sp. A9, a bacterium isolated from the Chernobyl exclusion zone [44].

For a better characterization of the high binding capacity, a DBM dependent study was performed with *Brachybacterium* sp. G1. Because the association process is relatively fast, an incubation time of 2 h was chosen. No significant increase of associated uranium (20 μM) was detected for DBM higher than 0.1 mg/mL (Fig 4C). Above this level sorption values increased from 76.1 ± 4.4% to 87.8 ± 0.7%. At low DBM (< 0.05 mg/mL) less than 50% of added uranium was associated. For corresponding experiments with *H*. *noricense*, an exposure time of 48 h was used, taking the slower and more complex association behavior into account. The obtained results show that DBM below 0.4 mg/mL are not sufficient to associate 50% of initial uranium. Biomass concentrations between 0.5 and 0.7 mg/mL result in the highest sorption values between 89.0 ± 0.2% and 91.0 ± 0.6%. At the higher DBM of 1.8 mg/mL a decrease to 75.3 ± 2.0% was recorded, indicating that the use of a large amount of biomass is not necessary. A reason for this is that cells can interact with each other and decrease the availability of potential binding sites [45, 46].

In conclusion, experiments with *Brachybacterium* sp. G1 were performed with a DBM of 0.075 mg/mL, but for studies with *H*. *noricense* the higher DBM of 0.5 mg/mL was necessary. A possible explanation for the high sorption capacity of *Brachybacterium* sp. G1 is the high number of carboxyl groups within the peptidoglycan layer of the cell wall [47]. The vast majority of these groups are deprotonated at pC_H+_ 6.0 and, therefore represent potential binding sites for the predominant positively charged uranyl species. The low DBM also promote a high sorption capacity.

### Microscopic investigation of uranium bioassociation

**Fig 5:**
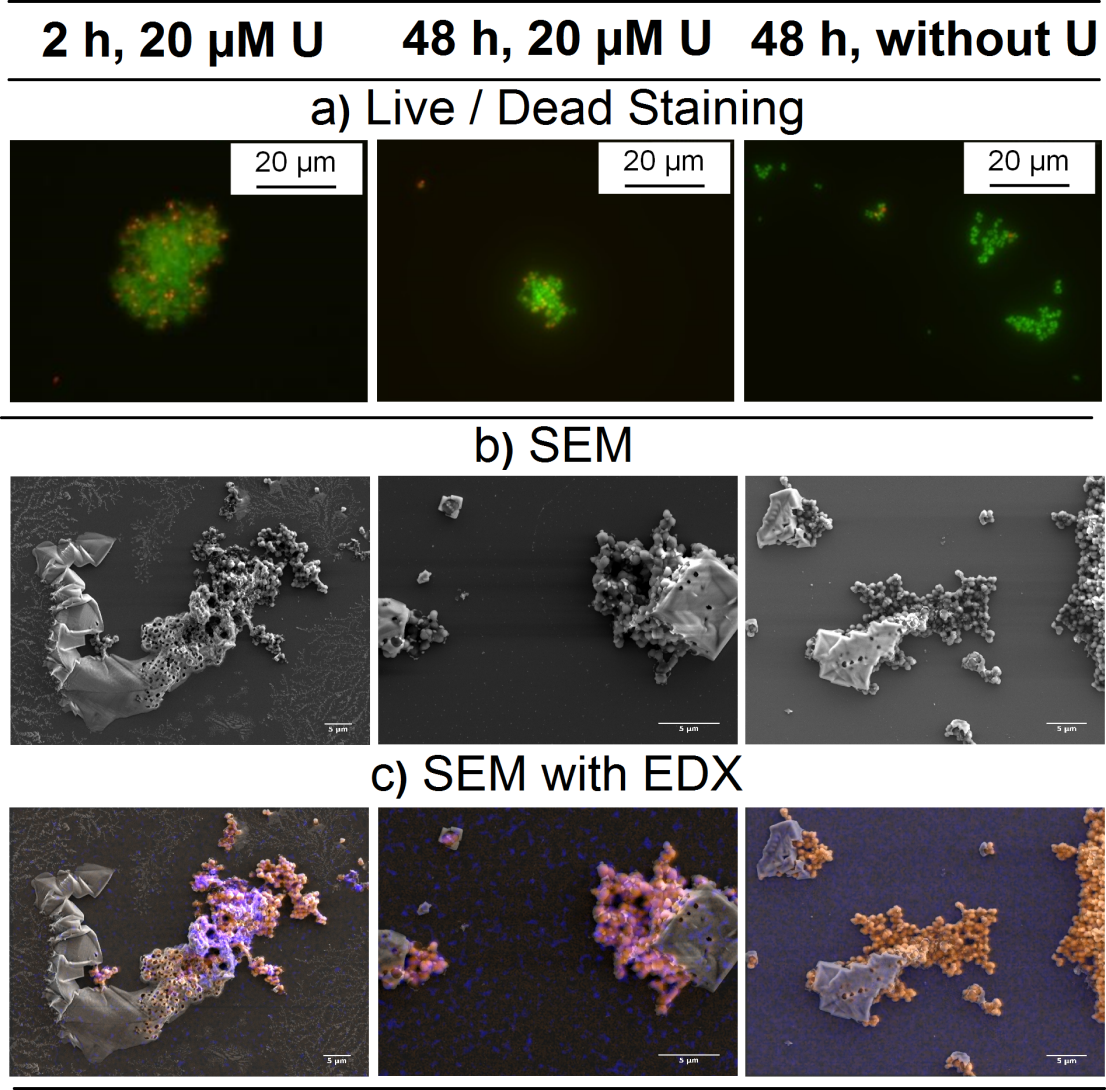
Micrographs of *Brachybacterium* sp. G1 incubated with and without uranium (20 μM U, pC_H+_ 6, 1.7 M NaCl) a) Fluorescence microscopy images of live/dead stained cells (green fluorescence–alive, red fluorescence– dead), b) Electron microscopy images (secondary electrons), c) Mapping of organic elements (orange, C, N, O) and uranium (blue). Scale bar on SEM images is 5 μm. SEM images are enlarged in SI (S2 Fig).

Cells incubated with an initial uranium concentration of 20 μM were investigated with live/dead staining and electron microscopy (Fig 5). From the results, it was determined that cell agglomeration was not induced by the presence of uranium, as the distribution regarding evenly dispersed and agglomerated cells did not change upon exposure to uranium. Rather, a change in distribution occurred due to growth phase. With increasing growth time, the amount of agglomerated cells increased. Cells of *Brachybacterium* sp. G1 used for uranium interaction studies were harvested in the late exponential phase, a possible explanation for the observed agglomeration. Additionally, agglomeration may have been a response to elevated salt concentration at the ionic strength used for the experiment (1.7 M). Nevertheless, uranium affected neither cell viability nor appearance within the first 48 h. In contrast, a significant change in cell appearance was observed for *H*. *noricense* [11]: huge agglomerates formed as a function of exposure time and uranium concentration.

For SEM experiments, no further sample preparations steps were performed and 1.7 M NaCl was used as background electrolyte. Hence, salt crystals are visible on SEM images. Elemental mapping by EDX showed the association of uranium with cells of *Brachybacterium* sp. G1. The strong EDX signal after 2 h confirmed the fast association process for this microorganism.

### Spectroscopic investigation of uranium bioassociation

ATR FT-IR spectroscopy has been proven to be very sensitive to reactions occurring at interfaces between water and solids of mineral or biological origin [11, 48, 49]. It provides *in situ*, but also time-resolved molecular information [35]. In a flow-through experiment, the absorption properties of an immobile phase, e.g. a microbial film, are directly monitored throughout the surface processes. Surface reactions can be identified in real time with a resolution in the sub-minute range from the pure bacteria up to complete bioassociation with the metal, e.g. U(VI) including all intermediate steps. The kinetic spectral information obtained might be complementary to results of other spectroscopic techniques mainly investigating batch samples.

**Fig 6:**
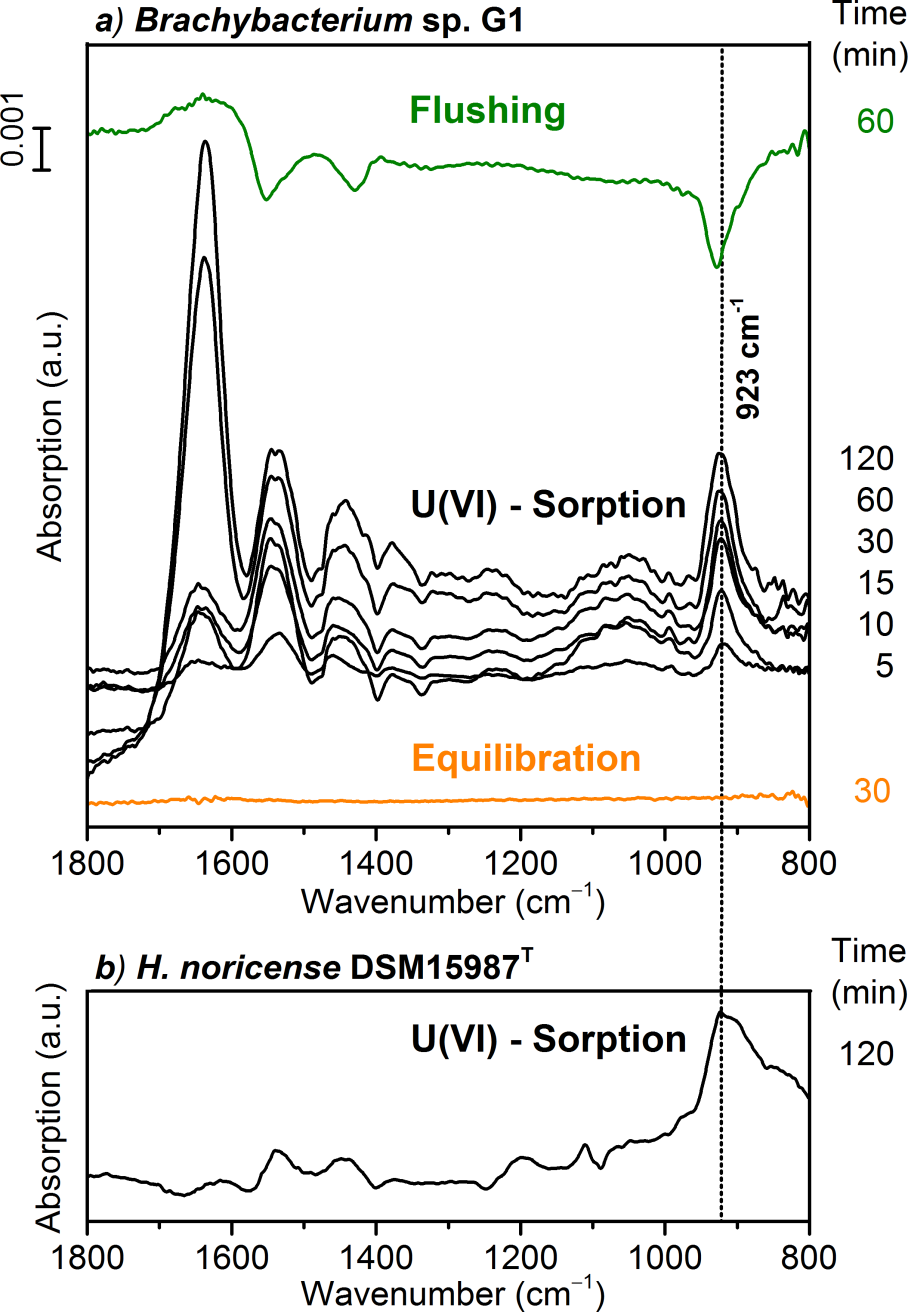
a) *In situ* ATR FT-IR difference spectra of U(VI) sorption on *Brachybacterium* sp. G1 cells ([U(VI)] = 40 μM, pC_H+_ 6, 1.7 M NaCl). The “Equilibration” spectrum confirms a stable bacterial film on the ATR crystal. “U(VI)—sorption” spectra were recorded at different times after induction of U(VI) association. “Flushing” shows the reversibility. b) For comparison the spectrum of U(VI) bioassociation on *H*. *noricense* cells after 120 min ([U(VI)] = 40 μM, pC_H+_ 6, 3 M NaCl) [11].

Prior to studying the bioassociation on *Brachybacterium* sp. G1 cells with *in situ* ATR FT-IR, the stability of the stationary microbial film on the ATR crystal´s surface was verified. The film was flushed with a blank solution for a prolonged time as an equilibration step. The difference spectrum obtained (Fig 6, red trace) showed no significant absorption changes in the spectral region from 1800 to 800 cm^-1^ within a time period of 30 min and therefore, indicates a sufficiently stable microbial film under the prevailing continuous flow conditions.

In general, we observed seven modes in the two investigated microorganisms, whereby six of them were occurring for the extremely halophilic archaeon and four for the moderately halophilic bacterium (Table 1). The IR spectra of the bioassociation process of *Brachybacterium* sp. G1 calculated from the spectra recorded before and after distinct times of U(VI) exposure are shown in Fig 6A (middle black traces). These spectra exhibit absorption bands with maxima at 1540, 1430 and 923 cm^-1^. The intensities of the bands increased during the two hours indicating U(VI) association with the bacterial film. After 120 min, no further intensity increase was observed, showing steady state conditions. For comparison, a corresponding spectrum of the archaeon *H*. *noricense* obtained at 120 min after U(VI) exposure is given in Fig 6B. A tentative assignment of the spectral features to functional groups of the microbial system is given in Table 1.

**Table 1:** Tentative assignment of infrared bands observed in difference spectra of *in situ* ATR FT-IR with *H*. *noricense* DSM15987^T^ ([U(VI)] = 40 μM, [NaCl] = 3 M, pC_H+_ 6) [11] and *Brachybacterium* sp. G1 ([U(VI) = 40 μM, [NaCl] = 1.7 M, pC_H+_ 6).

Bands with local maxima (cm^-1^)	Bands with local maxima (cm^-1^)	Tentative assignment to vibrational mode [50–53]
*H*. *noricense* [11]	*Brachybacterium* sp. G1	
-	1638	Amide-I
1535	1540	ν_as_ (COO^-^)/Amide-II
1435	1430	ν_s_ (COO^-^)
1197	-	ν_as_ (P-O)
1111	-	ν_as_ (P-O)
925	923	ν_3_ (UO_2_) coordinated to R–COO^−^
901	-	ν_3_ (UO_2_) coordinated to phosphoryl residues

Vibrational modes above 1200 cm^-1^ are observed for both microorganisms. In the spectra of *Brachybacterium* sp. G1, several bands are observed which can be attributed to general vibration modes of proteins such as amide-I and amide-II. These modes are usually observed around 1650 and 1550 cm^-1^ [50, 52].

Furthermore, contributions from carboxylate groups representing preferred binding sites of U(VI) species have to be considered at frequencies around 1550 and 1420 cm^-1^. Thus, in comparison to spectral data obtained from *H*. *noricense*, stronger contributions from protein modes are derived during the coordination of U(VI) to *Brachybacterium* sp. G1, especially after a sorption time above 60 min. The spectra clearly revealed an enormous increase in band intensity of the amide-I band after 60 min of U(VI) exposure, indicating a change in association behavior. It is conceivable that the spectra represent an uptake of U(VI) by the bacterium and subsequent enhanced interaction with intracellular proteins. This hypothesis needs to be investigated in more detail.

In the spectral range from 1200 to 1000 cm^-1^, bands mainly representing modes of phosphoryl functionalities but also of sugars residues, can be observed. In case of the archaeon *H*. *noricense*, distinct bands at 1197 and 1111 cm^-1^ correspond to the U(VI) interaction with phosphoryl groups [11, 50]. In contrast, for *Brachybacterium* sp. G1 no specific bands are observed, indicating no involvement of phosphoryl groups in U(VI) association. Thus, the broad band observed in the range from 1150 to 1000 cm^-1^ is more likely to indicate vibrational modes of sugar residues mainly of the cell wall. The peptidoglycan layers of *Brachybacterium* show a variety of anchored sugar molecules; for example galactose, glucose and glucosamine [19, 20]. Vibrational modes observed in the spectral region around 1000 cm^-1^ correspond to *ν*(C-O) stretching of the C-O-C glycosidic bond [53], which is present in high numbers in peptidoglycan. We assume that the polysaccharides, or more precisely the glycosidic bonds, do not serve as primary binding sites for U(VI) ions, but they are affected by the biosorption process of uranium leading to a signal in the respective IR spectra.

The absorption band between 950 and 900 cm^-1^ is assigned to the asymmetric stretching mode of the uranyl moiety *ν*_3_(*UO*_2_). In the spectrum of *Brachybacterium* sp. G1, this band shows a constant frequency maximum and bandwidth throughout the time interval of U(VI) exposure indicating the presence of a predominant species. The frequency of the band maximum at 923 cm^-1^ can be attributed to the coordination of uranyl ions to carboxylate functional groups [51, 52], and/or to aqueous species of U(VI), which also reveal a similar frequency in IR spectra under the prevailing conditions [54]. The spectrum of the subsequent flushing step showed negative bands at 1552, 1429 and 928 cm^-1^, representing the release of U(VI) species. These bands can be assigned to the antisymmetric and symmetric stretching modes of carboxylate groups and to the *ν*_3_(*UO*_2_) mode, respectively. The appearance of these bands during the release of uranyl species strongly suggests a coordination of uranyl species predominantly to carboxylate residues. This assumption is supported by the spectrum of *H*. *noricense*. The band of the *ν*_3_(*UO*_2_) mode shows an asymmetric shape and the 2^nd^ derivative indicates maxima at 925 and 905 cm^-1^, indicating an additional coordination of the uranyl species to phosphoryl groups as was found previously [11, 50, 52].

## Conclusions

A fundamental understanding of interactions of halophilic microorganisms with radionuclides and heavy metals is of high importance for the final storage of nuclear waste in salt rock. Therefore, the U(VI) interactions with a moderately halophilic bacterium as well as an extremely halophilic archaeon [11] were studied in detail. It was shown that the two investigated microorganisms interact with uranium in different ways. Uranium binds to *Brachybacterium* sp. G1 in the beginning in a pure biosorption process, meaning a fast removal from solution. *In situ* ATR FT-IR hinted a bioaccumulation after 1 h. In contrast the bioassociation with *H*. *noricense* is a multistage association process underlying different mechanisms [11]. The sorption capacity of both microorganisms differs significantly. For *Brachybacterium* sp. G1 maximal sorption capacities up to 971 ± 29 mg_U_/g_DBM_ were reached after 24 h with 40 μM U(VI), whereas *H*. *noricense* achieved merely 9.3 ± 0.4 mg_U_/g_DBM_ [11] after 14 d. Infrared spectroscopic studies showed the involvement of carboxylate groups in uranium association for both microorganisms, however phosphoryl interactions were only visible for *H*. *noricense*.

The bioassociation of uranium with cells of the two halophilic microorganisms is not only different with regard to the kinetics and the related mechanisms but also to the functional groups, which are involved within the first two hours, as studied by *in situ* ATR FT-IR spectroscopy. Because cells were harvested during late exponential phase and placed into a nutrient-free solution that did not promote growth, the possibility that cells were actively metabolizing was minimized. In this case, an important factor for biosorption is the cell wall structure, which differs significantly between the two investigated strains. The cell wall of the Gram-positive bacterium *Brachybacterium* sp. G1 consists of peptidoglycan layers with anchored teichoic acids and amino acids, like glutamic acid, glycine and alanine. The presence of glucosamine, galactose and glucose has also been shown [19, 20]. Next to potential sites for metal interactions, e.g. amino and hydroxyl functional groups, distinctly negatively charged sites like glutamic carboxyl and teichoic phosphodiester groups are crucial for metal sorption processes. Beveridge *et al*. [55] modified amine and carboxyl groups of the cell wall of the Gram-positive bacterium *Bacillus subtilis* to determine the dominant functional group in metal binding. From the modification of the carboxyl group it was concluded that carboxyl groups of glutamic acid represent the most potent binding sites. The predominant interaction of U(VI) with carboxylate and the absence of significant interactions of the actinide with phosphoryl groups demonstrated by *in situ* ATR FT-IR spectroscopy as well as the high content of glutamic acids in the cell wall of *Brachybacterium faecium* DSM4810^T^ [19] support this thesis.

In the case of bioassociation of uranium with *H*. *noricense*, a more complex mechanism was considered. Due to the multistage process, it was hypothesized that several different processes are involved. The cell membrane of the extremely halophilic archaeon *H*. *noricense* consists of derivatives of phosphatidylglycerol (PG), such as PG sulfate and PG methylphosphate, and S-layer proteins [17]. As described in the literature, the S-layer proteins of Haloarchaea are highly glycosylated, enriched with acidic residues and contain sulfated acid residues [18, 56]. Consequently, the infrared spectroscopic studies from the first two hours of bioassociation showed that in addition to carboxylate functional groups, phosphoryl groups are involved in the bioassociation process.

Both microorganisms are efficiently capable of immobilizing aqueous U(VI) in the lower micro molar range by association of about 80% within 48 h. However, both microorganisms interact in different ways with the actinide. *Brachybacterium* sp. G1 sorbs uranium within a short time frame on the cell surface and form a biocolloid, which can be further migrated. On the contrary *H*. *noricense* interact with uranium in multiple steps, which result in a potential formation of a biomineral [11]. The biomineralization of uranium implies the immobilization of uranium. Therefore, our study provides a detailed understanding of different interaction processes of halophilic bacteria and archaea indigenous to rock salt, which influences the migration behavior of uranium in a rock salt environment.

## Supporting information

S1 Fig Growth of *Brachybacterium* sp. G1 in dependence on pH value. Growth media was filtered Difco^TM^ Marine Broth 2216 (Becton, Dickinson and Company, United Sates) with a final NaCl concentration of 4.5% (0.8 M).
(PDF)

S2 Fig Electron microscopy images of *Brachybacterium* sp. G1 incubated with uranium (20 μM U, pC_H+_ 6, 1.7 M NaCl) for a) 2 h, b) 48 h and c) without uranium for 48 h. Mapping of organic elements (orange; C, N, O) and uranium (blue).
(PDF)
