# Supplementary material for: Comparative analysis of uranium bioassociation with halophilic bacteria and archaea
Source: PLoS One. 2018 Jan 12;13(1):e0190953. doi: 10.1371/journal.pone.0190953 (PMC5766140; doi:10.1371/journal.pone.0190953)
Supplement: S1 Fig — Growth media was filtered DifcoTM Marine Broth 2216 (Becton, Dickinson and Company, United Sates) with a final NaCl concentration of 4.5% (0.8 M). (PDF) [file pone.0190953.s001.pdf]

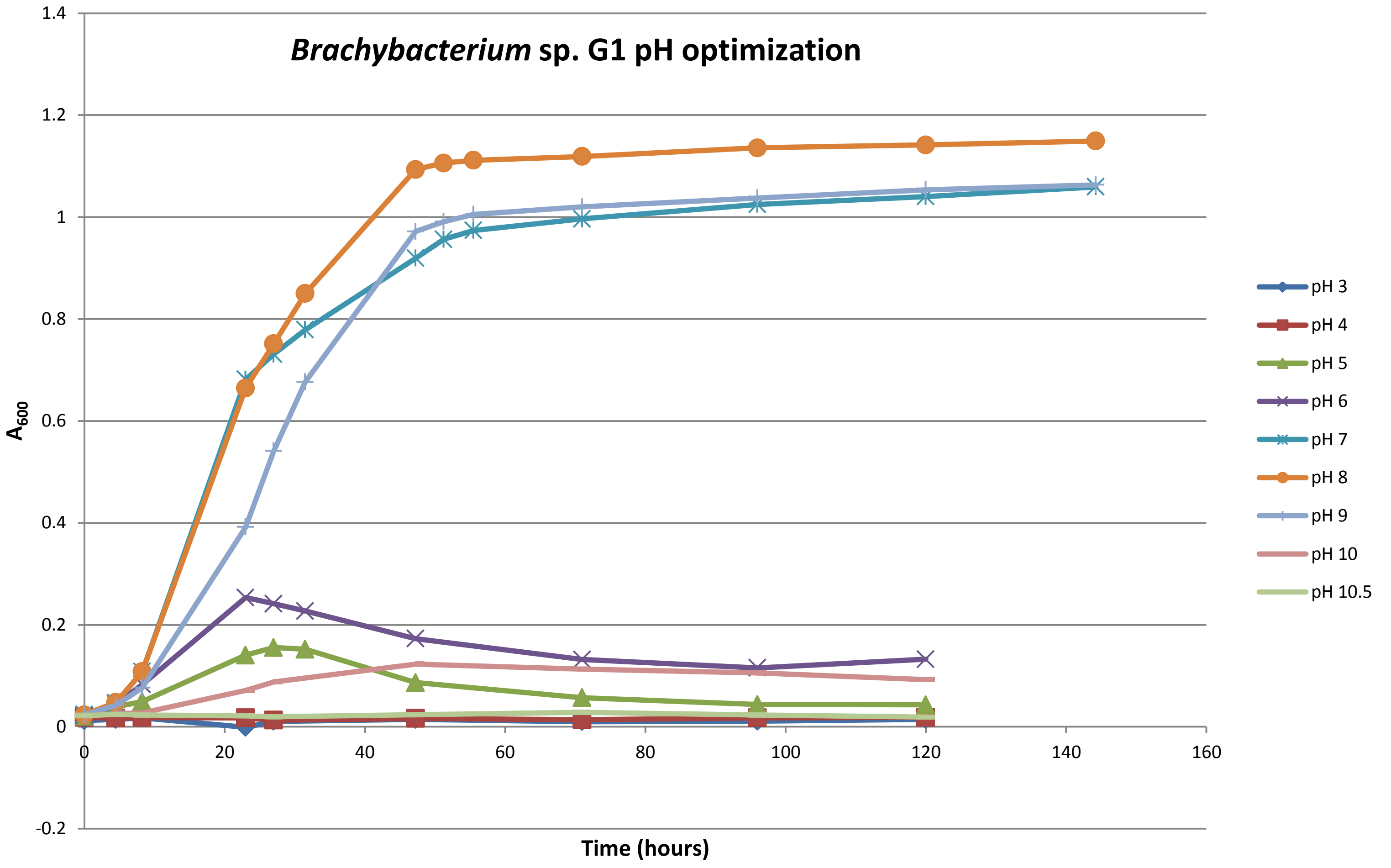

S1 Fig. Growth of *Brachybacterium* sp. G1 as a function of pH. Growth medium was filtered Difco™ Marine Broth 2216 (Becton, Dickinson and Company, United Sates) with a final NaCl concentration of 4.5% (0.8 M).
