# Supplementary material for: Comparative analysis of uranium bioassociation with halophilic bacteria and archaea
Source: PLoS One. 2018 Jan 12;13(1):e0190953. doi: 10.1371/journal.pone.0190953 (PMC5766140; doi:10.1371/journal.pone.0190953)
Supplement: S2 Fig — Electron microscopy images of Brachybacterium sp. G1 incubated with uranium (20 μM U, pCH+ 6, 1.7 M NaCl) for a) 2 h, b) 48 h and c) without uranium for 48 h. Mapping of organic elements (orange; C, N, O) and uranium (blue). (PDF) [file pone.0190953.s002.pdf]

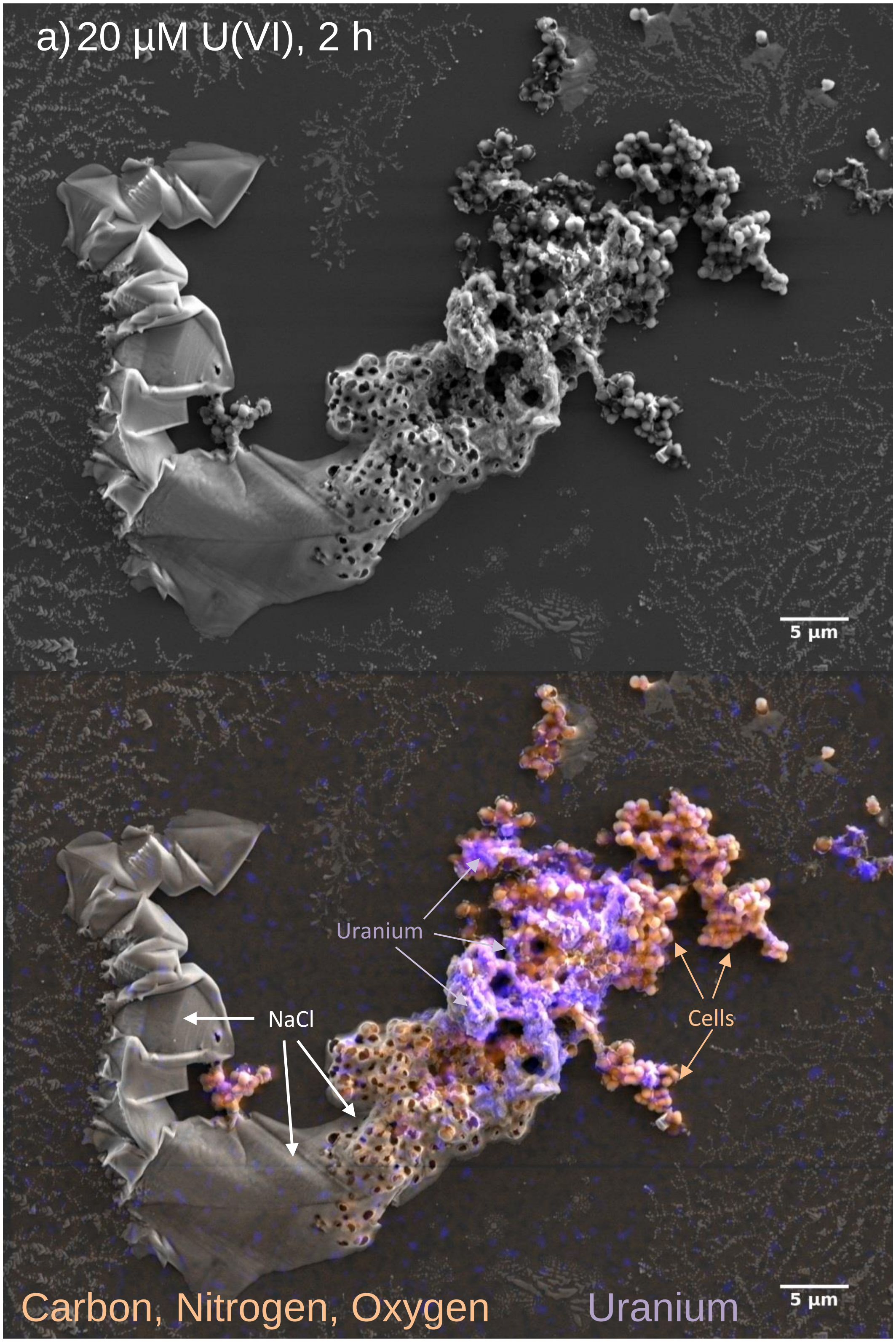

a) 20 µM U(VI), 2 h
5 µm
Uranium
NaCl
Cells
5 µm
Carbon, Nitrogen, Oxygen
Uranium

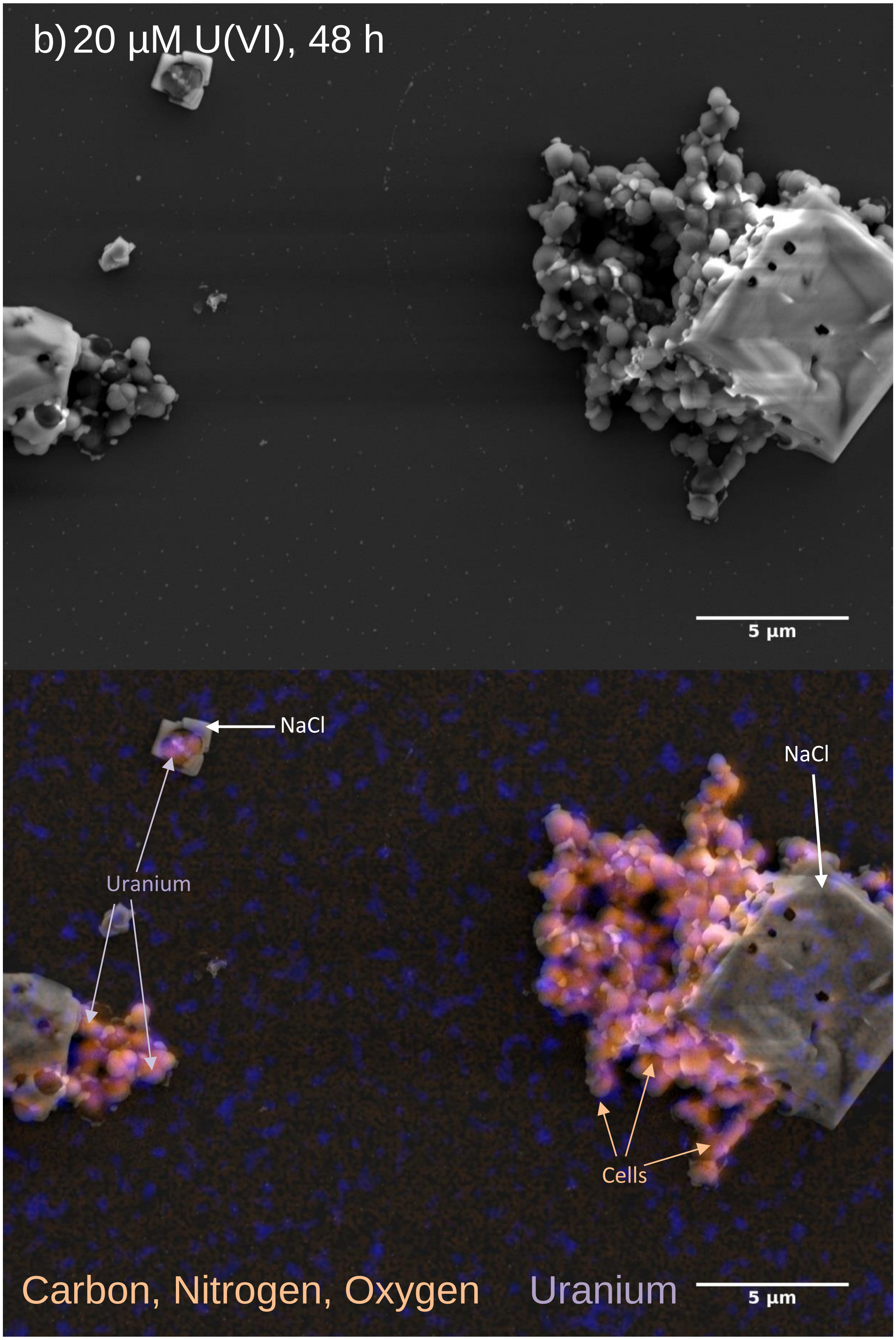

b) 20 µM U(VI), 48 h
5 µm
NaCl
NaCl
Uranium
Cells
Carbon, Nitrogen, Oxygen
Uranium
5 µm

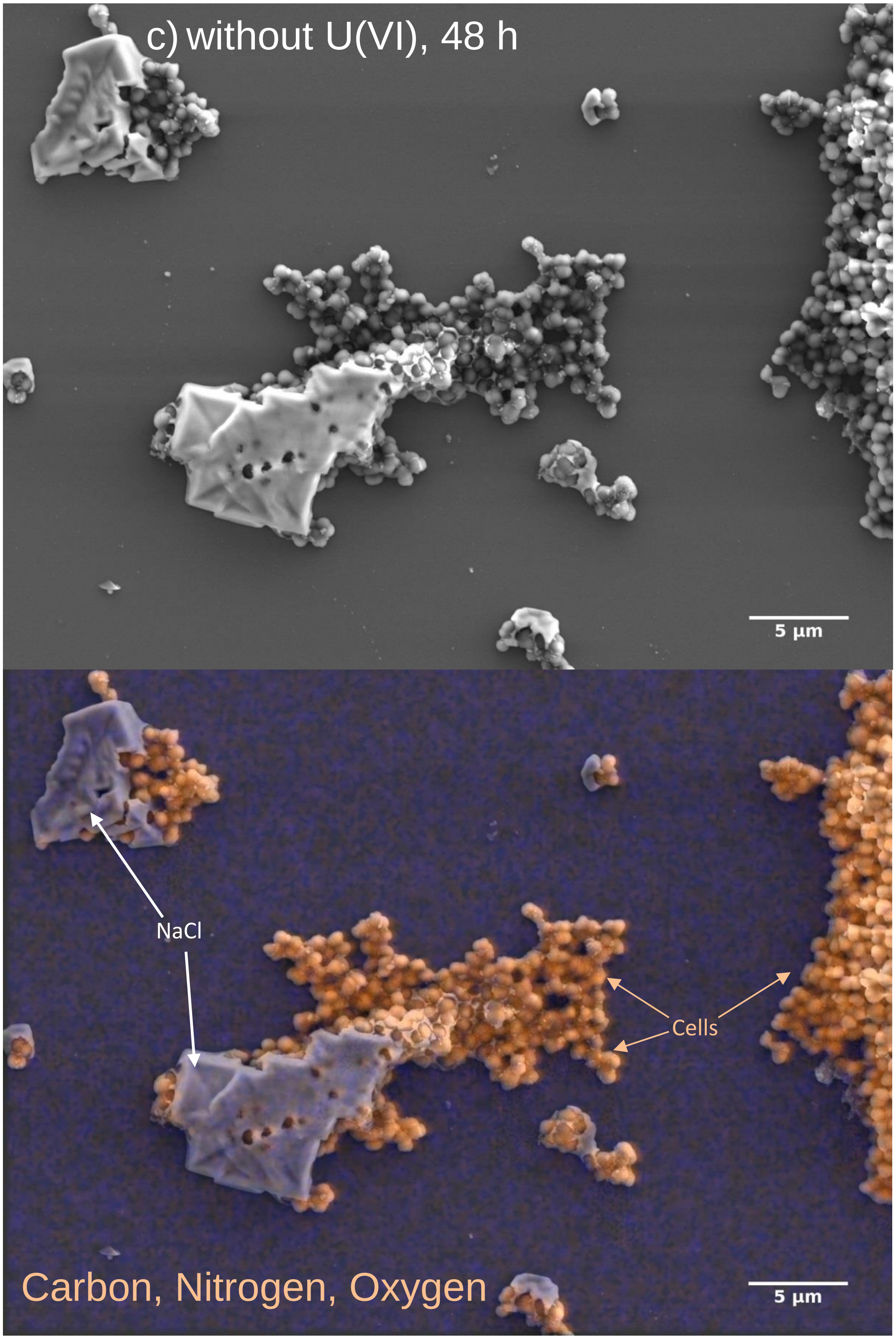

S2 Fig. Electron microscopy images of *Brachybacterium* sp. G1 incubated with uranium (20 µM U(VI), pC_{H+} 6, 1.7 M NaCl) for a) 2 h, b) 48 h and c) without uranium for 48 h. Mapping of organic elements (orange, C, N, O) and uranium (blue).
